# Expressive language and social communication abilities in children with spinal muscular atrophy type 1

**DOI:** 10.1111/dmcn.16461

**Published:** 2025-09-05

**Authors:** Chiara Brusa, Bianca Buchignani, Chiara Cutri, Giorgia Coratti, Elaine Clark, Emily Johnson, Nikki Cornell, Mariacristina Scoto, Marika Pane, Eugenio Maria Mercuri, Francesco Muntoni, Giovanni Baranello, The Language and Social Communication in SMA working group, Harriet Weststrate, Emily Barritt, Laura Antonaci, Daniela Leone, Concetta Palermo, Pinki Munot, Adnan Manzur

**Affiliations:** ^1^ UCL Great Ormond Street Institute of Child Health London UK; ^2^ Dubowitz Neuromuscular Centre Great Ormond Street Hospital for Children London UK; ^3^ Department of Developmental Neuroscience IRCCS Fondazione Stella Maris Pisa Italy; ^4^ Department of Translational Research and of New Surgical and Medical Technologies University of Pisa Pisa Italy; ^5^ Pediatric Neurology Università Cattolica del Sacro Cuore Roma Italy; ^6^ Centro Clinico Nemo Fondazione Policlinico Universitario Agostino Gemelli, IRCCS Roma Italy; ^7^ Neurodisability Service, Great Ormond Street Hospital for Children London UK; ^8^ Speech and Language Therapy Department Great Ormond Street Hospital for Children London UK; ^9^ National Institute for Health Research Great Ormond Street Hospital Biomedical Research Centre UCL Great Ormond Street Institute of Child Health London UK

## Abstract

**Aim:**

To investigate parent‐reported expressive language and social communication abilities in children with spinal muscular atrophy type 1 (SMA1) treated with disease‐modifying therapies.

**Method:**

This was a cross‐sectional feasibility study performed at the Dubowitz Neuromuscular Centre, London (UK), and the Centro Clinico Nemo Pediatrico, Rome (Italy), testing the use of the MacArthur‐Bates Communicative Development Inventories (MB‐CDIs, 8 months+) to explore vocabulary production, and the Social Communication Questionnaire (SCQ, 4 years+) to investigate social communication.

**Results:**

Fifteen participants completed the MB‐CDIs (age range 2 years 2 months–6 years 9 months). Thirteen out of the 15 acquired verbal skills, although with scores below normal ranges. Thirty‐seven completed the SCQ (age range 4 years 0 months–9 years 0 months). Four out of the 37 scored 11 or more, suggesting the need for further assessment for autism spectrum disorder. Three out of four had completed the MB‐CDIs and were among the children able to say the lowest number of words. Other areas of concern included routines/ritualized patterns of behaviour (14 out of 37) and hyperreactivity to sensory input (5 out of 37).

**Interpretation:**

Treated children with SMA1 can acquire verbal skills, although this can be delayed. A percentage of them also present with social communication difficulties, especially when expressive language is more severely affected. Further assessments for language and social communication are, therefore, recommended and large prospective studies warranted to better characterize the spectrum of these abilities in treated children with or at risk of SMA1.

**Abbreviations**
CHOP INTEND: Children's Hospital of Philadelphia Infant Test of Neuromuscular Disorders
DMTs: disease‐modifying therapies
MB‐CDIs: MacArthur‐Bates Communicative Development Inventories
PEG(−J): percutaneous endoscopic gastrostomy(−jejunostomy)
SCQ: Social Communication Questionnaire
SMA: spinal muscular atrophy
SMA1: spinal muscular atrophy type 1
SMN: survival motor neuron

**What this paper adds**

- Children with spinal muscular atrophy type 1 treated post‐symptomatically can acquire expressive language, although this can be delayed.
- A percentage of these children can also present with social communication difficulties.
- In the absence of disease‐specific tests, available questionnaires such as the MB‐CDIs and the SCQ may be used as screening tools to prompt further assessments for early communication difficulties.

Spinal muscular atrophy (SMA) is an autosomal recessive neuromuscular disorder caused by mutations in the *SMN1* gene on chromosome 5q, leading to reduced expression of the survival motor neuron (SMN) protein. This results in dysfunction and degeneration of α‐motor neurons in the spinal cord and brainstem with progressive atrophy and weakness of limb, trunk, respiratory, and bulbar muscles.^1^

Humans carry at least one copy of the paralogue, centromeric *SMN2* gene which modulates the SMA phenotype, as a small fraction of its transcripts (approximately 10%) are alternatively spliced to produce the full‐length SMN protein. Historically, SMA has been divided into different types based on the age at onset and the maximum motor function achieved in untreated patients: three main types with paediatric onset (types 1–3) and two less common types, one with antenatal onset and very severe phenotype (type 0) and one with adult onset and milder phenotype (type 4). SMA type 1 (SMA1) is the most frequent type of SMA (50–60% of incident SMA), characterized by onset before 6 months of life and inability to achieve independent sitting. In the pretreatment era, it was the second most common genetic cause of death in children because of rapidly progressive motor, respiratory, and bulbar deterioration with more than 90% mortality by 2 years of age.

Over the past few years, the natural history of SMA1 has radically changed because of advances in the multidisciplinary standards of care^2^, ^3^ and the approval of three disease‐modifying therapies (DMTs) that increase the SMN protein expression through distinct molecular mechanisms. These are two mRNA therapies enhancing the amount of full‐length SMN transcripts alternatively spliced from the *SMN2* gene, nusinersen (given intrathecally, approved by the US Food and Drug Administration in 2016 and European Medicines Agency in 2017) and risdiplam (given orally, approved by the Food and Drug Administration in 2020 and European Medicines Agency in 2021), and one gene transfer therapy, onasemnogene abeparvovec, introducing the *SMN* gene through an AAV9 vector (given intravenously, approved by the Food and Drug Administration in 2019 and European Medicines Agency in 2020).^4^

The improvement in the survival of children with SMA1 is rapidly increasing the disease prevalence globally. In addition, there is increasing evidence of new phenotypes, with children who have SMA1 acquiring functional abilities and developmental milestones—including unsupported sitting—that had never been achieved before.^5^ While these newly emerging phenotypes are being characterized in terms of survival, motor, respiratory, and bulbar function, less is known about other areas of functioning such as cognition, language, and social communication.^6^, ^7^, ^8^ In the pretreatment era, data about cognition and language have been limited to the milder SMA phenotypes 2 and 3.^9^ In SMA1, information about these domains was mainly based on non‐formal assessments, with only a few very recent studies on treated children with SMA1 using standardized outcome measures to evaluate language and global developmental profile.^10^, ^11^, ^12^, ^13^, ^14^

We conducted a cross‐sectional feasibility study that aimed to expand knowledge of expressive language and social communication abilities in children with SMA1 treated with any of the three approved DMTs by testing the use of two standardized, validated parent/carer‐reported measures.

## METHOD

### Study design and setting

This was a two‐centre, observational, cross‐sectional feasibility study performed at two tertiary Neuromuscular Centres, the Dubowitz Neuromuscular Centre, Great Ormond Street Hospital for Children, London (UK), and the Centro Clinico Nemo Pediatrico, Rome (Italy). The study procedures were conducted either in person during the clinical appointments as per the disease standards of care or by telephone/video calls.

### Participants

As this was a feasibility study, it was performed in a subgroup of children with SMA1 recruited over very short periods. Participants had to have a genetically confirmed diagnosis of SMA and a clinical diagnosis of SMA1 further classified according to disease severity expressed by age at symptom onset: in the first 2 to 4 weeks of life (type 1a), by 3 months of age (type 1b), and between 3 and 6 months of age (type 1c). They had to be treated with any of the three approved DMTs: nusinersen, risdiplam, or onasemnogene abeparvovec. They had to be at least 8 months old for the questionnaire exploring expressive language abilities, and at least 4 years old for the one investigating social communication abilities. Parents had to agree and have time to complete the questionnaires. At the UK Centre, participants were recruited among those who attended clinical appointments from March to September 2019 for the evaluation of expressive language abilities, and then from January to September 2022 for the evaluation of social communication abilities. At the Italian Centre, participants were recruited among those who attended clinical appointments from November 2023 to April 2024 for the evaluation of social communication abilities. Data on expressive language skills collected at the Italian Centre have already been published in a previous study.^10^ No other criteria were used to select the study participants and no control group was included (Figures S1 and S2).

The study was part of the ethically approved natural history study at the two participating centres which entails informed written consent to research and research‐related publications (R&D reference 11DN15, REC reference 13/LO/1748; and 26/05/2020 1894).

### Variables

The clinical notes of all participants were reviewed to collect the following additional data: age and sex; SMA1 type and number of *SMN2* copies; DMT(s) and age at initiation; respiratory status at the time of completion of the questionnaire (tracheostomy yes/no, non‐invasive ventilation yes/no and, if yes, nocturnal only/more than 16 hours per day); feeding status at the time of completion of the questionnaire (oral, or mixed oral and enteral, or nasogastric tube, or percutaneous endoscopic gastrostomy‐jejunostomy; PEG‐J); motor status at the time of completion of the questionnaire; independent sitting (yes/no); score on the last SMA motor scale administered before the completion of the questionnaire: the Children's Hospital of Philadelphia Infant Test of Neuromuscular Disorders (CHOP INTEND),^15^, ^16^ the Revised Hammersmith Scale,^17^ or the Vignos Lower Extremity scale.^18^

### Outcome measures

#### Expressive language abilities

To investigate expressive language abilities, we selected the MacArthur‐Bates Communicative Development Inventories (MB‐CDIs) – Words and Gestures, long form.^19^ The MB‐CDIs are a group of parent/carer‐reported measures aiming to capture information on children's developing abilities in early language, including vocabulary comprehension and production, gestures, and grammar. The measures have been adapted to numerous languages and have been extensively used worldwide for typically and non‐typically developing children.^20^, ^21^ They come in three main forms: the Words and Gestures form for children aged 8 to 18 months, the Words and Sentence form for children aged 16 to 30 months, and the third form (CDI‐III) for children aged 30 to 37 months. The Words and Gestures form includes two parts: ‘Early Words’, and ‘Actions and Gestures’. For our study, we focused on part one—‘Early Words’—of this form, specifically on the vocabulary checklist (section D). The vocabulary checklist investigates a child's understanding and production of 396 words that fall into 19 semantic categories (e.g. animal sounds, animal names, vehicles, toys, food and drink, body parts, etc.). The vocabulary checklist is completed by a parent/carer who checks one of three possible boxes for each individual word, indicating whether their child understands the word, understands and says the word, or understands and signs it. Words in the checklist are left blank if the parent does not think their child understands them. For our participants, we were only interested in the number of words the child was able to understand and say. If a child understands and says every word on the vocabulary checklist, they receive a maximum score of 396. Although the Words and Gestures form is intended for children aged 8 to 18 months, we administered it also to children older than 18 months considering the MB‐CDIs can be used with older, developmentally delayed children as already done in previous studies.^20^, ^22^, ^23^

#### Social communication abilities

To investigate social communication abilities, we selected the Social Communication Questionnaire (SCQ) – Lifetime form. The SCQ,^24^ formerly known as the Autism Screening Questionnaire,^25^ is a widely recognized, brief, 40‐item, parent/carer‐reported screening measure focusing on symptoms of autism spectrum disorder likely to be observed by a primary caregiver. Each item in the SCQ requires a dichotomous ‘yes’/‘no’ response, which is scored 1 for presence of abnormal behaviour and 0 for absence of abnormal behaviour/normal behaviour. The first item—‘Is she/he now able to talk using short phrases or sentences?’—is not scored but rather determines whether the following six items relating to abnormal language must be answered. Therefore, ‘verbal’ children can score a total of 0 to 39 points while ‘non‐verbal’ children can score a total of 0 to 33 points. The Lifetime version of the questionnaire was used in our study. It investigates the complete developmental history and asks respondents to indicate whether the behaviours have ever been present for questions 2 to 19 and whether the behaviours were present at age 4 to 5 years for questions 20 to 40 (or to consider behaviours in the previous 12 months if the child is not 4 years old yet). This questionnaire is intended for children whose chronological age is older than 4 years, provided their cognitive age is more than 2 years, but there is increasing interest in its use in younger children.^26^ As a screening tool, the SCQ was created to determine who should receive a more complete, formal diagnostic assessment for autism spectrum disorder, and it was originally validated with a cut‐off of at least 15 points. However, a low sensitivity has been reported when using this threshold, especially in younger children (age <8 years). Some researchers have proposed adjusting the threshold according to the age of the participants and the purpose of the study, with a lower cut‐off point of 12^27^ or 11^28^ to improve sensitivity and reduce the likelihood of false negatives. In accordance with this suggestion, we selected at least 11 as the cut‐off for our study.

### Statistical analysis

Owing to the limited sample size, descriptive statistics were used to summarize demographic and clinical features of the study participants. For the same reason, only raw data for both the MB‐CDIs and the SCQ were presented.

For the raw scores collected from the vocabulary checklist of the MB‐CDIs – Words and Gestures, no comparisons with population‐weighted norms were possible as most parents/carers completed the questionnaire when their children were beyond the intended age range for this instrument. However, to consider data in the context of expressive language growth curves for typically developing children, we interrogated Wordbank,^29^ an open database for MB‐CDIs results providing unweighted vocabulary norms for typically developing children up to 30 months of age. In this study, vocabulary norms for the Word and Sentences form – English version – were extracted from Wordbank on 11th September 2024.

## RESULTS

### Study population

The results of the MB‐CDIs – Words and Gestures (number of words produced) were available for 13 participants from the UK Centre. Two participants completing the SCQ at the Italian Centre had previously completed the MB‐CDI, and their scores were added to those obtained by the UK participants. The 15 participants had the following features: six males and nine females, seven with type 1b and eight with type 1c, age range 2 years 2 months to 6 years 9 months, all treated with nusinersen at the time of completion of the questionnaire but one who also received onasemnogene abeparvovec‐xioi.

The results of the SCQ – Lifetime form were available for 18 participants from the UK Centre and 19 from the Italian Centre (11 out of 15 who previously completed the MB‐CDIs and another 26 children). The 37 participants had the following features: 18 males and 19 females, five with SMA1a, 20 with SMA1b, and 12 with SMA1c, age range 4 years 0 months to 9 years 0 months, 30 out of 37 treated with nusinersen, 1 out of 37 switched from nusinersen to risdiplam, 2 out of 37 received onasemnogene abeparvovec‐xioi after nusinersen, 1 out of 37 received onasemnogene abeparvovec‐xioi only, 2 out of 37 received risdiplam after onasemnogene abeparvovec‐xioi, and 1 out of 37 received risdiplam only.

Clinical data are summarized in Table 1, and details for each participant, including sex, SMA1 subtype, number of *SMN2* copies, DMT(s) and age at initiation, age, respiratory, feeding, and motor status at the time of completion of the questionnaires, are reported in Table S1.

**TABLE 1:** Study sample.

Characteristic	Participants completing MB‐CDIs (*n* = 15)	Participants completing SCQ (*n* = 37)
Sex		
Male, *n* (%)	6 (40)	18 (49)
Female, *n* (%)	9 (60)	19 (51)
SMA1 subtype		
1a, *n* (%)	0	5 (13.5)
1b, *n* (%)	7 (47)	20 (54)
1c, *n* (%)	8 (53)	12 (32.5)
SMN2 copy number		
2, *n* (%)	14 (93)	32 (86.5)
3, *n* (%)	1 (7)	5 (13.5)
DMT		
First DMT, *n* (%)	Nusinersen 15 (100)	Nusinersen 33 (89)
		Onasemnogene abeparvovec 3 (8)
		Risdiplam 1 (3)
Shift to second DMT, *n* (%)	1 (7)	4 (11)
Age at first DMT in months, median (range)	9 (2–57)	8 (1–50)
MB‐CDIs data		
Age at completion in months, median (range)	33 (26–69)	
Ventilatory support	NIV > 16 hours/day, 2 (13)	
	n‐NIV 10 (67)	
	No support 3 (20)	
Feeding status	PEG(−J) 9 (60)	
	Nasogastric tube 1 (7)	
	Mixed PEG out of orally 1 (7)	
	Orally 4 (26)	
Independent sitting	Yes 9 (60)	
CHOP INTEND total score, mean (range)	42 (23–56)	
SCQ data		
Age at completion, median (range)		6 years 1 month (4 years 0 months–9 years 1 month)
Ventilatory support		Tracheostomy 4 (11)
		NIV > 16 h out of day 2 (5.5)
		n‐NIV 26 (70)
		If unwell only 3 (8)
		No support 2 (5.5)
Feeding status		PEG(−J) 22 (59)
		Nasogastric tube 1 (3)
		Mixed PEG out of orally 4 (11)
		Orally 10 (27)
Independent sitting		Yes 26 (70)
SMA scale total score, mean (range)		RHS (*n* = 26) 10 (3–27)
		Vignos scale (*n* = 4) 9.5 (9–10)
		n/a (*n* = 7)

Abbreviations: CHOP INTEND, Children's Hospital of Philadelphia Infant Test of Neuromuscular Disorders; DMT, disease‐modifying therapy; MB‐CDIs, MacArthur‐Bates Communicative Development Inventories; n/a, not administered/not assessed; NIV, non‐invasive ventilation; PEG(−J), percutaneous endoscopic gastrostomy(−jejunostomy); RHS, Revised Hammersmith Scale; SCQ, Social Communication Questionnaire; SMA, spinal muscular atrophy; SMN, survival motor neuron.

### Expressive language abilities

**FIGURE 1:**
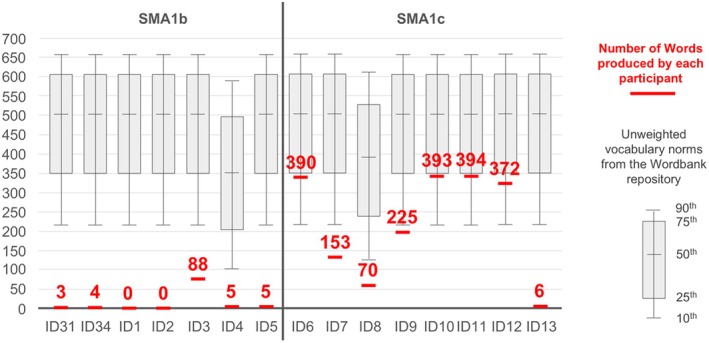
Scores obtained on the vocabulary checklist (‘understands and says’) of the MacArthur‐Bates Communicative Development Inventories – Words and Gestures. To consider the scores in the context of expressive language growth curves for typically developing children, we interrogated Wordbank, an open database providing unweighted vocabulary norms for children up to 30 months of age. For participants no older than 30 months (ID2 30 months, ID4 26 months, ID7 30 months, ID8 27 months, ID13 30 months) we used age‐matched unweighted norms while for those older than 30 months (ID31, ID34, ID1, ID3, ID5, ID6, ID9, ID10, ID11, ID12) we used unweighted norms for children aged 30 months. Abbreviation: SMA1a, SMA1b, spinal muscular atrophy types 1a, 1b.

The parents of the seven participants with SMA1b completed the MB‐CDIs – Words and Gestures when their children were between 2 years 2 months and 2 years 11 months of age. The median score on the vocabulary checklist (‘says’) was 4 out of 396 words (range 0–88): two children, ID1 and ID2, were unable to say any words; four children, ID31, ID34, ID4, and ID5, were only able to say three to five words; and one child was able to say 88 words (Figure 1). All seven children had two *SMN2* copies, they were all on non‐invasive ventilation (six nocturnal only, one for more than 16 hours per day), and all PEG fed except one who was orally fed, and they had a mean score at last CHOP INTEND of 43 out of 64 (range 24–52). They had started nusinersen at a median age of 6 months (range 2–10).

The parents of the eight participants with SMA1c completed the MB‐CDIs – Words and Gestures when their children were between 2 years 3 months and 6 years 9 months of age. The median score on the vocabulary checklist (‘says’) was 298 out of 396 words (range 6–394); one child, ID13, was able to say six words only (Figure 1). All children had two *SMN2* copies but one who had three. Three patients did not need ventilatory support, four were on nocturnal non‐invasive ventilation only, and one on non‐invasive ventilation for more than 16 hours per day. Four patients were orally fed with one of the four also requiring PEG support, three were PEG fed, and one was fed through a nasogastric tube. Their mean score at last CHOP INTEND was 41 out of 64 (range 23–56). They had started nusinersen at a median age of 14.5 months (range 8–57).

The MB‐CDIs results for each study participant are shown in Figure 1 and Table S1.

### Social communication abilities

**FIGURE 2:**
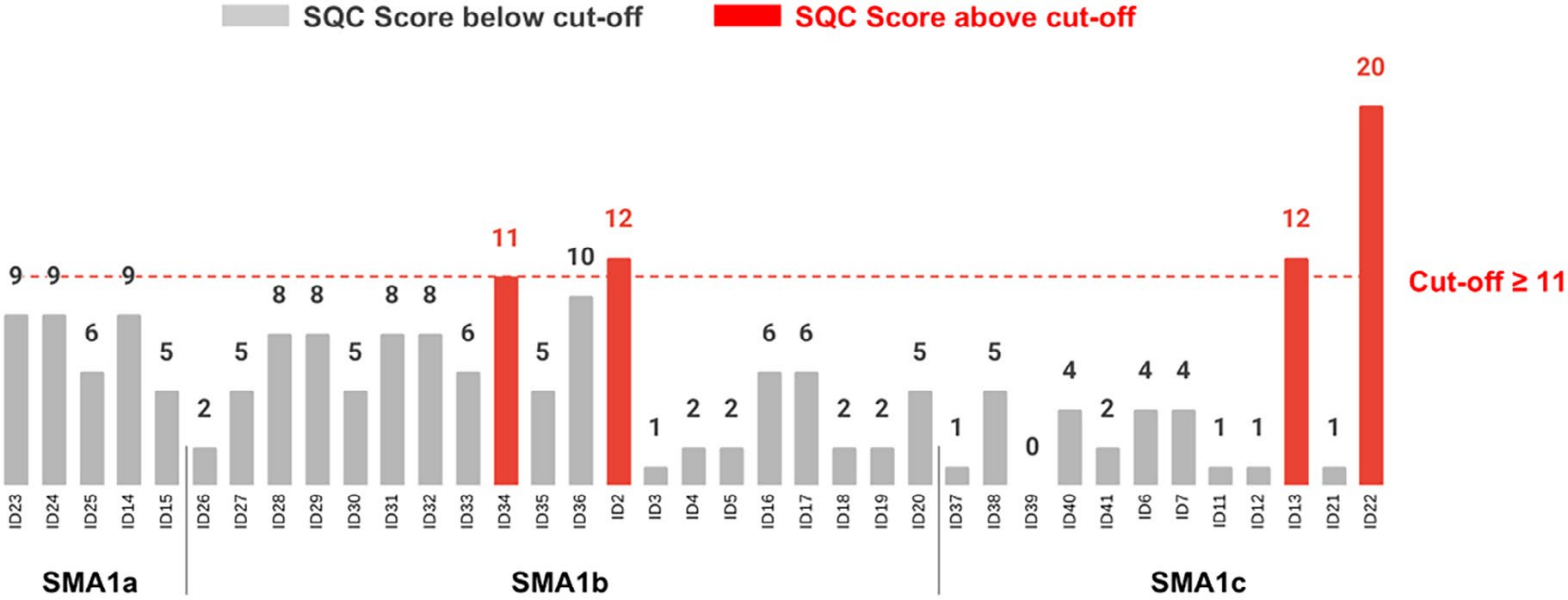
Scores obtained on the SCQ – Lifetime form. Abbreviations: SCQ, Social Communication Questionnaire; SMA1a, SMA1b, spinal muscular atrophy types 1a, 1b.

Four out of 37 participants whose parents/carers completed the SCQ, two children with SMA1b with two *SMN2* copies (ID34 and ID2) and two with SMA1c with two and three *SMN2* copies respectively (ID13 and ID22), reached or scored above the pre‐specified cut‐off of 11, suggesting the need for further assessment for autism spectrum disorder, as shown in Figure 2. At the time of the study, one of them (ID2) had a complete formal assessment confirming the diagnosis of autism spectrum disorder (moderate degree), one did not meet the criteria for autism spectrum disorder, and the remaining two had been referred to local services for assessment. The clinical features of the four participants who reached or scored above the SCQ cut‐off are summarized in Table 2.

**TABLE 2:** Clinical features of the participants who reached or scored above the SCQ cut‐off.

Pt ID	Sex	SMA1 sub‐type	SMN2 copies	First DMT, age at initiation	CHOP INTEND motor score at initiation of first DMT	Pt age at completion of SCQ	Ventilatory support at completion of SCQ	Feeding status at completion of SCQ	Sitting (yes/no) at completion of SCQ	Last CHOP INTEND motor score at completion of SCQ	Last SMA motor score at completion of SCQ	SCQ score (total = 39)	Routines yes/no	Hyperreactivity to sensory input (yes/no)	Number of questions not answered
ID34	M	1b	2	N, 2 months^a^	26 out of 64	4 years 7 months	n‐NIV	Orally	Yes	50 out of 64	RHS 18 out of 69	11	Yes	No	0
ID2	M	1b	2	N, 3 months	n/a	5 years 9 months	n‐NIV	PEG	No	49 out of 64^b^	RHS 4 out of 69	12	Yes	No	12
ID13	F	1c	2	N, 8 months	n/a	5 years 4 months	n‐NIV	PEG/oral	No	38 out of 64	RHS 3 out of 69	12	Yes	Yes	0
ID22	F	1c	3	OA, 10 months	37 out of 64	4 years 0 months	No	Orally	Yes	n/a	RHS 9 out of 69	20	No	Yes	0

Abbreviations: CHOP INTEND, Children's Hospital of Philadelphia Infant Test of Neuromuscular Disorders; DMT, disease‐modifying therapy; F, female; M, male; N, nusinersen; n/a, not administered/not assessed; n‐NIV, nocturnal non‐invasive ventilation; OA, onasemnogene abeparvovec; PEG, percutaneous endoscopic gastrostomy; Pt ID, participant identity; RHS, Revised Hammersmith Scale; SCQ, Social Communication Questionnaire; SMA, spinal muscular atrophy; SMN, survival motor neuron.

^a^ Patient ID34 also received onasemnogene abeparvovec at 24 months of age.

^b^ Completed at 2 years 10 months of age.

When administering the SCQ questionnaire to parents/carers, we observed a noteworthy percentage of participants (12 out of 37, 32%) reporting the presence of inflexible adherence to routines or ritualized patterns of behaviour (question 8): patients ID34, ID2, and ID13 as well as another nine children who did not reach the SCQ cut‐off (three with SMA1a, three with SMA1b, and three with SMA1c). Restrictive, repetitive patterns of behaviour were reported by parents/carers especially during bedtime, while watching cartoons, or in relation to caring procedures. In addition, we observed a noteworthy percentage of participants (5 out of 37, 13%) with reported hyperreactivity to sensory input (questions 14 and 37): one child with SMA1a showing adverse response to noise, one with SMA1b, and three with SMA1c not responding positively to social touch (i.e. being touched and/or having their wheelchair being touched or, in general, being approached by another child).

Twenty‐one out of 37 parents/carers were unable to answer one or more questions owing to their child being non‐verbal and/or not strong enough to perform the action(s) included in the SCQ (e.g. ‘When she/he was 4 to 5, did she/he ever talk with you just to be friendly [rather than to get something]?’, ‘Has she/he ever had any complicated movements of her/his whole body, such as spinning or repeatedly bouncing up and down?’, ‘When she/he was 4 to 5, did she/he play cooperatively in games that required joining in with a group of other children, such as hide‐and‐seek or ball games?’). So, in these cases, those items could not contribute to the total score.

The SCQ results for each study participant are shown in Figure 2 and Table S1.

### SCQ scores in children who obtained low scores on the MB‐CDIs

Three of the four patients who reached or scored above the SCQ cut‐off (ID34, ID2, and ID13) were among the children obtaining the lowest scores on the MB‐CDIs vocabulary checklist as they were reported to be able to say four, zero, and six words respectively. Patient ID22, who obtained the highest SCQ score, did not complete the MB‐CDI. Among the four other patients obtaining very low scores on the MB‐CDIs vocabulary checklist—five words or fewer—ID1 did not complete the SCQ whereas ID31, ID4, and ID5 did not reach the SCQ cut‐off. Despite this, patients ID31 and ID4 were both reported to have ritualized patterns of behaviour.

## DISCUSSION

This two‐centre cross‐sectional feasibility study explored expressive language and social communication abilities of treated children with SMA1 from a parent/carer perspective by testing the use of well‐known, widely used, standardized assessments.

For expressive language skills, 86% (13 out of 15) of parents/carers reported their child was able to say three words or more. However, 7 out of 13 obtained very low raw scores. The results clearly showed that children with SMA1c were able to say more words than those with SMA1b (median score 298 for those with SMA1c versus 4 for SMA1b). These two groups had similar results for CHOP INTEND scores at the time of completing the questionnaire but a higher number of patients in the SMA1c group needed no respiratory or feeding support compared with the SMA1b group. Comparing these scores with normative standards was not possible as the MB‐CDIs – Words and Gestures was completed when most participants were older than the validated age range. However, most scores were below the unweighted vocabulary norms available from the MB‐CDIs Wordbank repository. These results expand the findings from a recent study that reported longitudinal MB‐CDIs data in a smaller cohort of 24 children with SMA1 and 12 infants identified by newborn screening.^10^ Additional evidence comes from three other recent studies using general neurodevelopmental scales. A longitudinal study on seven pre‐symptomatic and 11 post‐symptomatic children with SMA1 assessed with the Bayley Scales of Infant and Toddler Development, Third Edition^11^ showed that a significant proportion of patients treated post‐symptomatically had below‐average scores on communicative (as well as cognitive) subscales, with better outcomes observed in pre‐symptomatic children. A cross‐sectional study of 15 children with SMA1 treated post‐symptomatically and assessed with the Griffiths Scales of Child Development, Third Edition^12^ showed normal range scores in the Language and Communication (as well as the Foundation of Learning) subscales, although with mean scores in the borderline range and general development quotients corresponding to a global developmental delay. Finally, a newborn screening project assessing 40 children with SMA treated in the first weeks of life with the Bayley Scales of Infant and Toddler Development, Third Edition^13^ showed overall normal language development with no dependence on *SMN2* copy number. However, in this last study, lower cognitive development scores were observed in children with lower numbers of *SMN2* copies despite all children with two *SMN2* copies being treated in the first weeks of life.

For social communication skills, 11% of the infants in our cohort reported behaviours suggesting the need for further assessment for autism spectrum disorder. These results should be interpreted with caution as more than half of the parents/carers could not answer one or more questions owing to the SCQ not considering SMA1‐specific verbal and/or motor difficulties. We explored the possible impact of different variables on social communication abilities. No univocal findings emerged on the impact of early vocabulary and bulbar function. Three of the four patients reaching or scoring above the SCQ cut‐off were able to say no or a very limited number of words on the MB‐CDI, and they were either orally or PEG fed, while the fourth patient was fully orally fed but had not completed the MB‐CDIs so, unfortunately, we had no data on their early vocabulary. Three patients had two copies of the *SMN2* gene and one had three *SMN2* copies. However, the interpretation of the impact of *SMN2* copies is limited by the small number of participants carrying three *SMN2* copies in our SMA1 cohort which is line with data from the literature.

This study contributes to delineating further the new challenges presented by treated children with SMA1 and provides preliminary evidence for further larger‐scale projects. Our findings highlight the need to identify or develop tools in which items are not affected by severe motor impairment. The lack of such tools is partly responsible for the little evidence on this domain that is currently available in the literature for SMA. Clinicians are becoming increasingly aware of potential neurodevelopmental difficulties in children with SMA1, as discussed during a recent workshop on multi‐system involvement in SMA^30^ and in a recent international survey on co‐occurring neurodevelopmental conditions in early‐onset SMA.^31^ However, a better understanding of the prevalence of individual neurodevelopmental comorbidities and of the impact of motor impairment as well as other factors, such as ventilation needs and cognitive difficulties, is required.^32^

The MB‐CDIs is considered a relevant instrument in generating knowledge on child language, particularly in special populations; however, its validity as an early screening tool for language difficulties was recently questioned^21^ because of some methodological shortcomings identified in previous studies such as the mere explorative nature of the study, the focus on expressive language only, the heterogeneity of measures used as reference tests, the generally small sample sizes, the presence of spectrum and/or verification biases, and the rarity of reported confidential intervals. As for the SCQ, although it has been used for other disorders associated with verbal and/or motor difficulties such as cerebral palsy,^33^ it was not standardized specifically for children with severe motor impairment and, in our experience, seems to have a limited use in the subgroup of children with SMA1 with limited motor abilities. Nonetheless, in the absence of more disease‐specific tests, we believe that questionnaires such as the MB‐CDIs and the SCQ may be used as screening tools to identify early communication difficulties because they are quick and easy to administer; however, their results should be interpreted with caution and should be the first step to prompt access to neurodisability services. Careful consideration should be given to limitations in motor abilities that could be misinterpreted as social communication impairment or to the overall level of severity of the phenotype that may be responsible for weakness in the phonatory muscles with subsequent reduction of the number of words detected. Because of this, especially when abnormal findings are detected, both questionnaires should be accompanied by cognitive and neuropsychological standardized tests to delineate the neurodevelopmental profile of each child with SMA1 more accurately, and by a complete speech and language therapy review performed alongside to better interpret results from the MB‐CDI. These are all crucial aspects considering that earlier detection and intervention can lead to better outcomes for children with neurodevelopmental disorders.^34^

Raising awareness on potential neurodevelopmental impairments in children with SMA1 is essential to design future studies aiming to further clarify the role of the SMN protein on early brain development. This is becoming especially important now that more children are treated early after birth by the implementation of newborn screening programmes, but concerns about neurodevelopmental difficulties still persist for those carrying two copies of the *SMN2* gene. Future research on these domains would help to identify new outcome measures to investigate the efficacy of current and future therapeutic strategies for SMA. The use of augmentative alternative communication or other tools such as eye‐tracking devices should also be considered as alternative options for the subgroup of patients with higher motor impairment.

## FUNDING INFORMATION

SMA REACH UK project. Biogen. Muscular Dystrophy UK, the MRC Translational Research Centre at UCL and Newcastle, and the National Institute for Health Research Biomedical Research Centre at Great Ormond Street Hospital for Children NHS Foundation Trust and University College London.

## CONFLICT OF INTEREST STATEMENT

B.B. was partially supported by the Italian Ministry of Health (GRANT RC 2024) to IRCCS Stella Maris Foundation. GC reports participation on advisory boards and steering committees for Biogen, Roche, and Novartis. EJ has received speaker's fees/an honorarium from Roche and Biogen. NC has received an honorarium from Roche. MS reports participation to scientific advisory boards and teaching initiatives for Novartis Gene Therapies, Biogen, and Roche; is involved as principal investigator in clinical trials for Novartis Gene Therapies, Biogen, Roche; additionally is Co‐Principal Investigator of the SMA REACH UK study. MP reports personal fees from Biogen, PTC Therapeutics, AveXis, Sarepta, outside the submitted work. EM reports personal fees for advisory boards, steering committee, speaker fees, or consultancies from Biogen, Roche, Avexis and/orNovartis; and is supported by RF‐2019‐12370334 (Italian Health Ministry). FM reports participation on scientific advisory boards and teaching initiatives for AveXis, Biogen, Roche, and Novartis. GB is principal investigator of clinical trials sponsored by Pfizer, NS Pharma, and Reveragen; has received speaker's and/or consulting fees from Sarepta, PTC Therapeutics, Biogen, Novartis Gene Therapies (AveXis), and Roche; and has worked as principal investigator of SMA studies sponsored by Novartis Gene Therapies and Roche. The other authors have stated that they had no interests that might be perceived as posing a conflict or bias.

## Supporting information


**Figure S1:** Recruitment for the MB‐CDI.

**Figure S2:** Recruitment for the SCQ.

**Table S1:** Clinical features of each study participant at completion of the MB‐CDI and the SCQ.

## Data Availability

The data that support the findings of this study are available from the corresponding author upon reasonable request.
