# Supplementary material for: Expressive language and social communication abilities in children with spinal muscular atrophy type 1
Source: Dev Med Child Neurol. 2025 Sep 5;68(5):696–705. doi: 10.1111/dmcn.16461 (PMC13056017; doi:10.1111/dmcn.16461)
Supplement: Supplementary file 1 — Figure S1: Recruitment for the MB‐CDI. [file DMCN-68-696-s001.pdf]

UK Centre (total n=73 children with SMA1)

↓

- age 8 months+
- attending appointment from March to September 2019
- agree to / time to complete the MB-CDI

↓

13 participants
+ 2 participants who completed the SCQ at the Italian Centre and had previously completed the MB-CDI
