# Supplementary material for: Expressive language and social communication abilities in children with spinal muscular atrophy type 1
Source: Dev Med Child Neurol. 2025 Sep 5;68(5):696–705. doi: 10.1111/dmcn.16461 (PMC13056017; doi:10.1111/dmcn.16461)
Supplement: Supplementary file 2 — Figure S2: Recruitment for the SCQ. [file DMCN-68-696-s003.pdf]

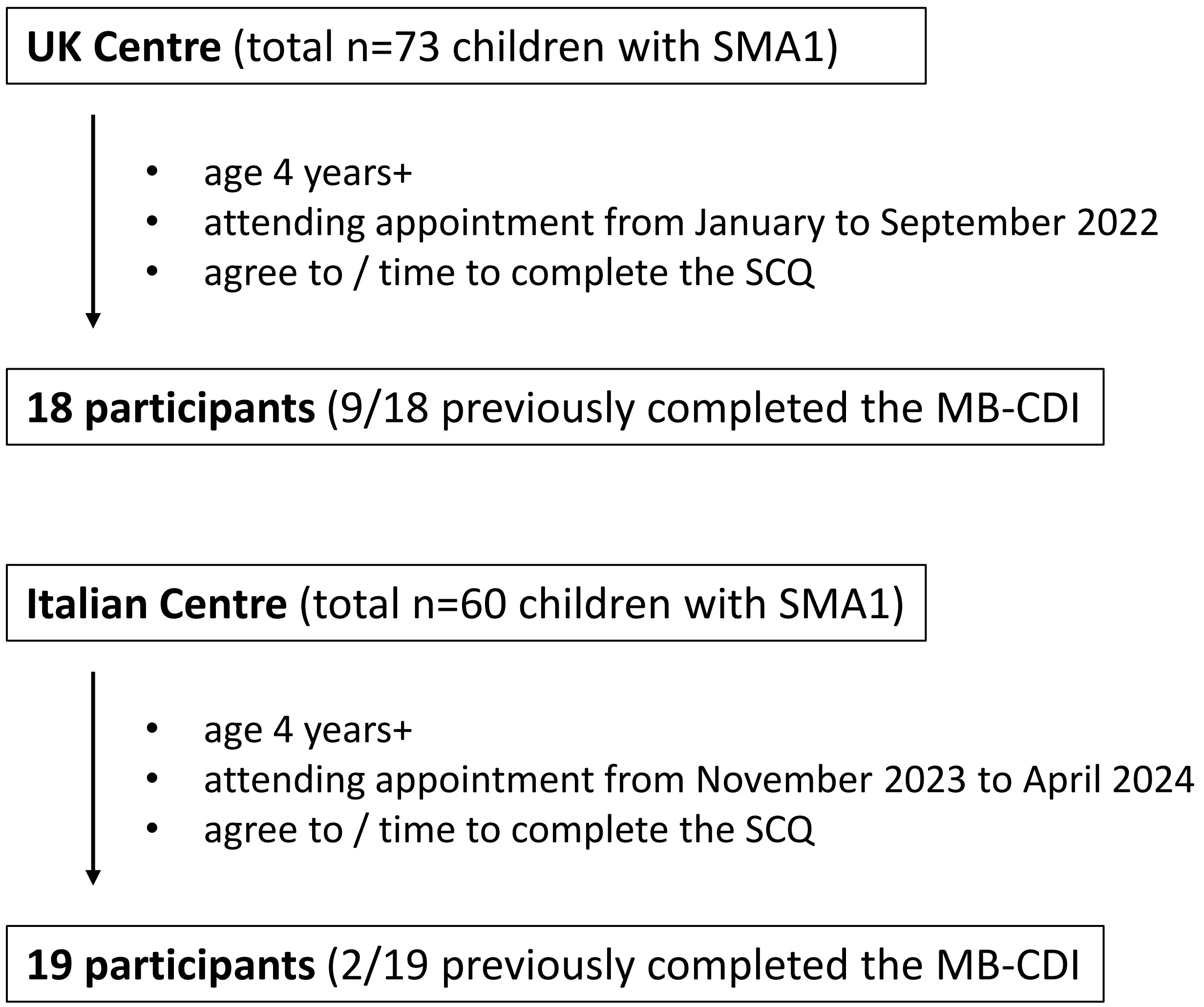
UK Centre (total n=73 children with SMA1)
age 4 years+
attending appointment from January to September 2022
agree to / time to complete the SCQ
18 participants (9/18 previously completed the MB-CDI
Italian Centre (total n=60 children with SMA1)
age 4 years+
attending appointment from November 2023 to April 2024
agree to / time to complete the SCQ
19 participants (2/19 previously completed the MB-CDI
