# Supplementary material for: Expressive language and social communication abilities in children with spinal muscular atrophy type 1
Source: Dev Med Child Neurol. 2025 Sep 5;68(5):696–705. doi: 10.1111/dmcn.16461 (PMC13056017; doi:10.1111/dmcn.16461)
Supplement: Supplementary file 3 — Table S1: Clinical features of each study participant at completion of the MB‐CDI and the SCQ. [file DMCN-68-696-s002.docx]

**Supplementary Table 1**. Clinical features of each study participant at completion of the MB-CDI and the SCQ

| **Pt ID** | **Sex** | **SMA1 sub-type** | **SMN2 copies** | **First disease modifying therapy, age at initiation** | **MB-CDI pt age** | **MB-CDI ventilatory support** | **MB-CDI feeding status** | **MB-CDI sitting yes/no** | **MB-CDI**  **last CHOP INTEND motor score** | **MB-CDI number of words produced** | **SCQ**  **pt age** | **SCQ**  **ventilatory support** | **SCQ**  **feeding status** | **SCQ**  **sitting yes/no** | **SCQ**  **last SMA motor score** | **SCQ score (total = 39)** | **Routines yes/no** | **Hyper reactivity to sensory input yes/no** | **No. of not answered questions** |
| --- | --- | --- | --- | --- | --- | --- | --- | --- | --- | --- | --- | --- | --- | --- | --- | --- | --- | --- | --- |
| ID1 | M | 1b | 2 | N, 9m | 2y 9m | n-NIV | PEG | yes | 52/64 | 0 | n/a | n/a | n/a | n/a | n/a | n/a | n/a | n/a | n/a |
| ID2 | M | 1b | 2 | N, 3m | 2y 6m | n-NIV | PEG | no | 51/64 | 0 | 5y 9m | n-NIV | PEG | no | RHS 4/69 | **12** | **yes** | no | **12** |
| ID3 | F | 1b | 2 | N, 10m | 2y 11m | n-NIV | PEG | yes | 43/64 | 88 | 5y 11m | n-NIV | PEG | yes | RHS 6/69 | 1 | no | no | 2 |
| ID4 | M | 1b | 2 | N, 4m | 2y 2m | n-NIV | PEG-J | yes | 48/64 | 5 | 4y 10m | n-NIV | PEG | yes | RHS 25/69 | 2 | **yes** | **yes** | 0 |
| ID5 | M | 1b | 2 | N, 10m | 2y 9m | NIV>16h/d | PEG | no | 24/64 | 5 | 5y 8m | NIV>16h/d | PEG | no | n/a | 2 | no | no | 21 |
| ID6 | F | 1c | 2 | N, 24m | 4y 2m | n-NIV | orally | yes | 42/64 | 390 | 6y 11m | n-NIV | orally | yes | RHS 5/69 | 4 | **yes** | **yes** | 2 |
| ID7 | F | 1c | 2 | N, 8m | 2y 6m | NIV>16h/d | NG tube | no | 36/64 | 153 | 4y 11m | n-NIV | NG tube | yes | Vignos 9/10 | 4 | no | no | 0 |
| ID8 | F | 1c | 2 | N, 9m | 2y 3m | n-NIV | PEG | no | 41/64 | 70 | n/a | n/a | n/a | n/a | n/a | n/a | n/a | n/a | n/a |
| ID9 | F | 1c | 2 | N, 57m | 6y 9m | n-NIV | PEG | no | 23/64 | 225 | n/a | n/a | n/a | n/a | n/a | n/a | n/a | n/a | n/a |
| ID10 | F | 1c | 2 | N, 11m | 3y 5m | no | PEG/oral | yes | 47/64 | 393 | n/a | n/a | n/a | n/a | n/a | n/a | n/a | n/a | n/a |
| ID11 | F | 1c | 2 | N, 20m | 3y 10m | no | orally | yes | 56/64 | 394 | 7y 3m | if unwell | orally | yes | RHS 16/69 | 1 | **yes** | no | 0 |
| ID12 | M | 1c | 3 | N, 18m | 3y 4m | no | orally | yes | 50/64 | 372 | 6y 2m | If unwell | orally | yes | RHS 7/69 | 1 | **yes** | no | 1 |
| ID13 | F | 1c | 2 | N, 8m | 2y 6m | n-NIV | PEG | no | 44/64 | 6 | 5y 4m | n-NIV | PEG/oral | no | RHS 3/69 | **12** | **yes** | **yes** | **0** |
| ID14 | M | 1a | 2 | N, 1m | n/a | n/a | n/a | n/a | n/a | n/a | 4y 9m | n-NIV | PEG-J | yes | RHS 9/69 | 9 | no | no | 6 |
| ID15 | M | 1a | 2 | N, 50m | n/a | n/a | n/a | n/a | n/a | n/a | 9y 0m | trachy | PEG | no | Vignos 10/10 | 5 | **yes** | **yes** | 22 |
| ID16 | M | 1b | 2 | N, 16m | n/a | n/a | n/a | n/a | n/a | n/a | 6y 5m | NIV>16h/d | PEG | no | Vignos 10/10 | 6 | no | no | 21 |
| ID17 | M | 1b | 2 | N, 3m | n/a | n/a | n/a | n/a | n/a | n/a | 5y 1m | n-NIV | PEG/oral | yes | Vignos 9/10 | 6 | no | no | 8 |
| ID18 | F | 1b | 2 | N, 2m | n/a | n/a | n/a | n/a | n/a | n/a | 5y 1m | no | PEG/oral | yes | RHS 10/69 | 2 | no | no | 1 |
| ID19 | M | 1b | 2 | N, 2m | n/a | n/a | n/a | n/a | n/a | n/a | 4y 3m | n-NIV | PEG | yes | RHS 8/69 | 2 | no | no | 0 |
| ID20 | F | 1b | 2 | N, 2m * | n/a | n/a | n/a | n/a | n/a | n/a | 4y 4m | n-NIV | PEG-J | yes | RHS 8/69 | 5 | no | no | 1 |
| ID21 | M | 1c | 3 | N, 19m | n/a | n/a | n/a | n/a | n/a | n/a | 7y 0m | If unwell | orally | yes | RHS 22/69 | 1 | no | no | 0 |
| ID22 | F | 1c | 3 | OA, 10m | n/a | n/a | n/a | n/a | n/a | n/a | 4y 0m | no | orally | yes | RHS 9/69 | **20** | no | **yes** | **0** |
| ID23 | F | 1a | 2 | N, 9 m | n/a | n/a | n/a | n/a | n/a | n/a | 6y 1m | n-NIV | orally | yes | 54/64 | 9 | **yes** | no | 0 |
| ID24 | M | 1a | 2 | OA, 3 m ** | n/a | n/a | n/a | n/a | n/a | n/a | 5y 9m | n-NIV | PEG | yes | 40/64 | 9 | no | no | 5 |
| ID25 | M | 1a | 2 | N, 6 m | n/a | n/a | n/a | n/a | n/a | n/a | 5y 7m | trachy | PEG | no | 3/64 | 6 | **yes** | no | 8 |
| ID26 | F | 1b | 2 | N, 41 m | n/a | n/a | n/a | n/a | n/a | n/a | 8y 0m | n-NIV | PEG | no | 25/64 | 2 | no | no | 2 |
| ID27 | F | 1b | 2 | N, 30 m | n/a | n/a | n/a | n/a | n/a | n/a | 9y 1m | n-NIV | PEG | no | 13/64 | 5 | no | no | 8 |
| ID28 | F | 1b | 2 | N, 3 m | n/a | n/a | n/a | n/a | n/a | n/a | 7y 11m | n-NIV | PEG | yes | 52/64 | 8 | no | no | 0 |
| ID29 | M | 1b | 2 | N, 6 m | n/a | n/a | n/a | n/a | n/a | n/a | 7y 6m | n-NIV | PEG | yes | 43/64 | 8 | **yes** | no | 0 |
| ID30 | F | 1b | 2 | R, 6 m | n/a | n/a | n/a | n/a | n/a | n/a | 7y 10m | n-NIV | PEG | yes | 35/64 | 5 | no | no | 4 |
| ID31 | F | 1b | 2 | N, 6 m | 2y 9m | n-NIV | PEG | yes | 41/64 | 3 | 4y 9m | n-NIV | PEG | yes | 45/64 | 8 | **yes** | no | 2 |
| ID32 | F | 1b | 2 | N, 24 m | n/a | n/a | n/a | n/a | n/a | n/a | 8y 4m | trachy | PEG | no | 15/64 | 8 | no | no | 6 |
| ID33 | F | 1b | 2 | N, 6 m | n/a | n/a | n/a | n/a | n/a | n/a | 8y 4m | n-NIV | PEG | yes | 41/64 | 6 | no | no | 3 |
| ID34 | M | 1b | 2 | N, 2 m *** | 2y 8m | n-NIV | orally | yes | 50/64 | 4 | 4y 7m | n-NIV | orally | yes | 50/64 | **11** | **yes** | no | 0 |
| ID35 | M | 1b | 2 | OA, 2 m ^ | n/a | n/a | n/a | n/a | n/a | n/a | 5y 8m | n-NIV | orally | yes | 54/64 | 5 | no | no | 0 |
| ID36 | F | 1b | 2 | N, 35 m | n/a | n/a | n/a | n/a | n/a | n/a | 7y 10m | trachy | PEG | no | 8/64 | 10 | **yes** | no | 4 |
| ID37 | M | 1c | 3 | N, 26 m | n/a | n/a | n/a | n/a | n/a | n/a | 8y 11m | n-NIV | PEG/oral | no | 27/64 | 1 | no | no | 0 |
| ID38 | M | 1c | 3 | N, 4 m | n/a | n/a | n/a | n/a | n/a | n/a | 7y 3m | n-NIV | PEG | yes | 42/64 | 5 | no | no | 5 |
| ID39 | M | 1c | 2 | N, 9 m | n/a | n/a | n/a | n/a | n/a | n/a | 6y 7m | n-NIV | orally | yes | 52/64 | 0 | no | no | 0 |
| ID40 | F | 1c | 2 | N, 6 m | n/a | n/a | n/a | n/a | n/a | n/a | 5y 5m | n-NIV | orally | yes | 54/64 | 4 | no | no | 0 |
| ID41 | F | 1c | 2 | N, 9 m | n/a | n/a | n/a | n/a | n/a | n/a | 7y 1m | n-NIV | PEG | yes | 42/64 | 2 | **yes** | no | 0 |

* Patient ID20 also received onasemnogene abeparvovec at 51 months of age

**Patient ID24 also received risdiplam (age at initiation not known)

***Patient ID34 also received onasemnogene abeparvovec at 24 months of age

^ Patient ID35 also received risdiplam starting from 30 months of age

Abbreviations: Pt = participant; MB-CDI = MacArthur-Bates Communicative Development Inventory; SCQ = Social Communication Questionnaire; d = day; m = months; y = years; N = nusinersen; OA = onasemnogene abeparvovec; R = risdiplam; n-NIV = nocturnal non-invasive ventilation; trachy = tracheostomy; PEG(-J) = percutaneous endoscopic gastrostomy(-jejunostomy); NG tube = nasogastric tube; CHOP INTEND = Children's Hospital of Philadelphia Infant Test of Neuromuscular Disorders; RHS = Revised Hammersmith Scale; Vignos scale 9/10 = in a wheelchair; Vignos scale 10/10 = confined to bed; n/a = not administered/not assessed
